# Incidence and Risk Factors for Breakthrough Invasive Mold Infections in Acute Myeloid Leukemia Patients Receiving Remission Induction Chemotherapy

**DOI:** 10.1093/ofid/ofz176

**Published:** 2019-04-12

**Authors:** Heena P Patel, Anthony J Perissinotti, Twisha S Patel, Dale L Bixby, Vincent D Marshall, Bernard L Marini

**Affiliations:** 1 Department of Pharmacy Services and Clinical Sciences, Michigan Medicine and the University of Michigan College of Pharmacy, Ann Arbor, Michigan; 2 Department of Internal Medicine, Division of Hematology & Oncology, Michigan Medicine, Ann Arbor, Michigan

**Keywords:** acute myeloid leukemia, antifungal prophylaxis, *Aspergillus*, azole antifungals, echinocandins, invasive fungal infections

## Abstract

**Background:**

Despite fungal prophylaxis, invasive mold infections (IMIs) are a significant cause of morbidity and mortality in patients with acute myeloid leukemia (AML) receiving remission induction chemotherapy. The choice of antifungal prophylaxis agent remains controversial, especially in the era of novel targeted therapies. We conducted a retrospective case–control study to determine the incidence of fungal infections and to identify risk factors associated with IMI.

**Methods:**

Adult patients with AML receiving anti-*Aspergillus* prophylaxis were included to determine the incidence of IMI per 1000 prophylaxis-days. Patients without and with IMI were matched 2:1 based on the day of IMI diagnosis, and multivariable models using logistic regression were constructed to identify risk factors for IMI.

**Results:**

Of the 162 included patients, 28 patients had a possible (n = 22), probable, or proven (n = 6) diagnosis of IMI. The incidence of proven or probable IMI per 1000 prophylaxis-days was not statistically different between anti-*Aspergillus* azoles and micafungin (1.6 vs 5.4, *P* = .11). The duration of prophylaxis with each agent did not predict IMI occurrence on regression analysis. Older age (odds ratio [OR], 1.04; 95% confidence interval [CI], 1.004–1.081; *P* = .03) and relapsed/refractory AML diagnosis (OR, 4.44; 95% CI, 1.56–12.64; *P* = .003) were associated with IMI on multivariable analysis.

**Conclusions:**

In cases that preclude use of anti-*Aspergillus* azoles for prophylaxis, micafungin 100 mg once daily may be considered; however, in older patients and those with relapsed/refractory disease, diligent monitoring for IMI is required, irrespective of the agent used for antifungal prophylaxis.

## Teaser

The risk for proven or probable breakthrough invasive mold infections (IMI) in acute myeloid leukemia (AML) patients undergoing induction chemotherapy was not different between patients receiving micafungin or anti-aspergillus azoles. Older patients and those with relapsed or refractory AML were at the highest risk of breakthrough IMI.

Despite recent advances in fungal prophylaxis, invasive mold infections (IMIs) remain a significant cause of morbidity and mortality in patients with acute myeloid leukemia (AML) undergoing remission–induction chemotherapy (RIC) [1, 2]. There is a general consensus that antifungal prophylaxis should be utilized in patients with AML; however, the choice of prophylaxis agent remains highly controversial [3].

Cornely et al. demonstrated a mortality benefit with anti-*Aspergillus* prophylaxis with posaconazole in AML patients undergoing RIC compared with fluconazole/itraconazole [4]. Unfortunately, drug–drug interactions and liver function abnormalities often limit use of anti-*Aspergillus* azole antifungals in this setting. In these situations, patients may be switched to echinocandins, which have fungistatic coverage against *Aspergillus* spp [2]. Evidence supporting the use of echinocandin prophylaxis in AML patients receiving RIC is limited, and as a result its use receives a lower-grade recommendation in both National Comprehensive Cancer Network and Infectious Diseases Society of America guidelines [5, 6].

Recently, Gomes et al. demonstrated a higher rate of invasive fungal infections (IFIs) and mortality in patients with AML receiving RIC who received echinocandin-based prophylaxis when compared with anti-*Aspergillus* azoles [7]. However, more recent analyses in various hematology populations have suggested no difference in IFI rates between echinocandin prophylaxis and anti-*Aspergillus* azoles [8, 9]. Given the discordance in study results, there remains a concern for increased risk of breakthrough IFIs in patients receiving echinocandin prophylaxis, and additional literature is needed to assess the safety and efficacy of echinocandins in AML patients receiving RIC. The purpose of our study was to compare the efficacy of echinocandin prophylaxis with anti-*Aspergillus* azole antifungals in preventing IMI in patients with AML during the period of neutropenia secondary to RIC. We also evaluated risk factors for IMI in this population.

## METHODS

### Patients

This retrospective study was conducted at Michigan Medicine, University of Michigan, Ann Arbor, Michigan, a 1000-bed tertiary care university-affiliated hospital with approximately 48 800 admissions annually. Ethics approval was obtained from the Institutional Review Board (IRB) at Michigan Medicine (approval number HUM00135806). Written informed consent was waived by the IRB given the retrospective nature of the study.

Adult (age ≥18 years) patients admitted to Michigan Medicine between June 2014 and August 2017 with a diagnosis of AML who received anti-*Aspergillus* prophylaxis (micafungin, voriconazole, or posaconazole) after induction therapy were considered for inclusion in this study. Micafungin was dosed at 100 mg intravenously once daily, posaconazole delayed-release tablet at 300 mg orally (PO) once daily, and voriconazole at 200 mg PO twice daily, dose-adjusted to a goal trough of 1.0–5.5 ng mL^-1^. Patients were identified using an internal leukemia database and the electronic medical record (EMR). Patients were excluded if they did not receive remission induction chemotherapy at Michigan Medicine or if they were not followed as inpatients at Michigan Medicine after induction chemotherapy. Patients who received nonintensive induction chemotherapy (eg, hypomethylating agents) were excluded. Patients were followed until their absolute neutrophil count (ANC) recovered and they no longer required anti-*Aspergillus* prophylaxis or discharge, whichever came first. Study data were collected using the EMR and managed using the Research Electronic Data Capture (REDCap) tools hosted at Michigan Medicine [10].

### Incidence Density of Invasive Mold Infections

The primary outcome was the incidence of proven or probable invasive mold infection (ppIMI), as defined by the European Organization for Research and Treatment of Cancer/Invasive Fungal Infections Cooperative Group and the National Institute of Allergy and Infectious Diseases Mycoses Study Group (EORTC/MSG) [11]. We collected days of anti-*Aspergillus* prophylaxis for all eligible patients post-RIC until the patients recovered their ANC or until patient discharge. Incidence density was calculated by dividing the absolute number of IMIs by the total number of IMI prophylaxis-days for each agent, based on the method proposed by Gomes et al. [7]. The IMIs were attributed to the agents that were used for a minimum of 96 hours in the 14 days preceding IMI diagnosis. The date of IMI diagnosis was the date on which imaging consistent with an IMI that satisfied EORTC/MSG criteria was obtained. The incidence was reported as the rate per 1000 prophylaxis-days (PPD). The incidence of proven, probable, or possible invasive mold infection (pppIMI) was also captured as a secondary outcome.

### Risk Factor Analysis

Baseline characteristics collected for all patients included patient age, gender, type of AML (newly diagnosed or relapsed/refractory), induction chemotherapy regimen, baseline ANC, duration of neutropenia, history of diabetes before chemotherapy, history of COPD, and smoking status. Additional risk factors collected included antifungal prophylaxis exposure, ICU admission, ICU length of stay, and prolonged hyperglycemia (blood glucose ≥200 g dL^-1^ for ≥96 hours). Control patients who did not develop an IMI were matched 2:1 to cases of pppIMI based on time from RIC initiation to the same day post-RIC as the day of IMI diagnosis in case patients. Antifungal prophylaxis exposure was defined as the use of a specific anti-*Aspergillus* prophylaxis agent for at least 96 hours in the 14 days preceding the diagnosis of pppIMI. After patients were matched, antifungal prophylaxis exposure data and risk factors were collected on non-IMI patients for a matching 14-day period (Supplementary Figure 1).

### Statistical Analyses

Statistical analyses were performed using SPSS software, version 24.0 (SPSS, Inc., Chicago, IL), and R, version 3.5.1 (R Foundation for Statistical Computing, Vienna, Austria). Dichotomous variables were analyzed using the Fisher exact test or Pearson chi-square test, and normally distributed continuous variables were analyzed using a 2-tailed Student *t* test. Non–normally distributed continuous variables were compared using the Mann-Whitney *U* test. Exploratory unconditional logistic regression analysis was performed to evaluate variables associated with pppIMI. Variables with a *P* value ≤.2 on univariable analysis were considered for inclusion in the multivariable model. For colinear variables with a *P* value ≤.2 on univariable analysis, only 1 variable was included in the multivariable analysis. A receiver operating characteristic (ROC) curve was generated to assess the sensitivity and specificity of the model. Multivariable logistic regressions were used to predict pppIMI occurrence based on the duration of prophylaxis with each of the antifungal agents. Duration of prophylaxis distributions were described with box and whisker plots, and LOESS smoothing with 95% confidence bands and rug plots was used to demonstrate increased risk of pppIMI with longer duration of therapy. A sensitivity analysis on the data determined the detectable effect size at 80% power in our primary hypothesis test (G*Power, 3.1.9.2). A *P* value of ≤.05 was considered statistically significant, and all *P* values were based on 2-tailed tests.

## RESULTS

**Figure 1.**
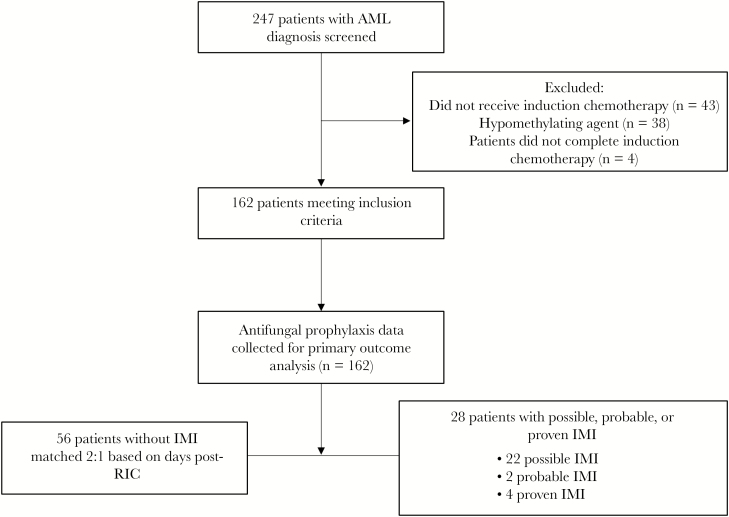
Patient enrollment diagram. Abbreviations: AML, acute myeloid leukemia; IMI, invasive mold infection; RIC, remission–induction chemotherapy.

A total of 247 patients with AML diagnosis were identified from the leukemia database and EMR. Eighty-five patients were excluded due to the aforementioned exclusion criteria. Of the 162 included patients, 6 (3.7%) patients had ppIMI and 28 (17.3%) patients had pppIMI (Figure 1). The median time to IMI from the start of RIC was 20 days. Days of antifungal prophylaxis were collected on all patients who met the inclusion criteria and are presented in Table 1. The total duration of antifungal prophylaxis by agent was 1702 days of voriconazole, 1374 days of posaconazole, and 554 days of micafungin. Three ppIMIs occurred in patients on posaconazole, 2 on voriconazole, and 3 on micafungin. The corresponding number of pppIMIs was 12 for posaconazole, 15 for voriconazole, and 12 for micafungin. The PPD rate of ppIMIs was 2.2 in the posaconazole group, 1.2 in the voriconazole group, and 5.4 in the micafungin group. The PPD rate of pppIMI was 8.7 in the posaconazole group, 8.8 in the voriconazole group, and 21.7 in the micafungin group. There was no statistically significant difference in the PPD rate of ppIMI between anti-*Aspergillus* azole prophylaxis and micafungin (1.6 vs 5.4, *P* = .11). The results of the sensitivity analysis showed that the minimum detectable effect size at 80% power was smaller than the actual difference in ppIMI found between the 2 groups. However, the PPD rate of pppIMIs was significantly different between patients receiving anti-*Aspergillus* azole prophylaxis and those receiving micafungin prophylaxis (8.45 vs 21.7, *P* = .01).

**Table 1.** Invasive Mold Infection per 1000 Prophylaxis-Day Rate

Agent	PPD Rate for ppIMI (Confidence Interval)	*P* Value	PPD Rate for pppIMI (Confidence Interval)	*P* Value
Anti-*Aspergillus* azoles	1.6 (1.0–4.0)		8.45 (6.0–12.0)	
Posaconazole	2.2 (0.01–6.0)		8.7 (5.0–15.0)	
Voriconazole	1.2 (0.01–4.0)		8.8 (5.0–14.0)	
Micafungin	5.4 (1.8–15.8)		21.7 (11.0–38.0)	
Anti-*Aspergillus* azole vs micafungin		.11		.01

The Fisher exact test was used to compare the PPD rates.

Abbreviations: PPD, per prophylaxis-day; ppIMI, proven or probable invasive mold infection; pppIMI, proven, probable, or possible invasive mold infection.

The 28 patients with pppIMI were matched 2:1 to 56 patients without IMI, assessing risk factors based on same-day postinduction chemotherapy of IMI diagnosis in the IMI group. Baseline characteristics of these 2 groups are presented in Table 2. The majority of patients in the IMI group had relapsed or refractory AML (64.3%) compared with only 30.4% of patients in the non-IMI group (*P* = .005). There was no significant difference in induction chemotherapy regimens between the IMI and non-IMI patients. Median baseline ANC was 0.15 × 10^9^ L^-1^ in the IMI group and 1.3 × 10^9^ L^-1^ in the non-IMI group (*P* = .14). There was no difference with regards to bone marrow transplant status before induction chemotherapy, ICU admission, ICU length of stay, or prolonged hyperglycemia between the 2 groups.

**Table 2.** Risk Factors for Invasive Mold Infection

Variable	Univariable Analysis			Multivariable LR	
	IMI (n = 28)	Non-IMI (n = 56)	*P* Value	OR (95% CI)	*P* Value
Median age (range), y	58 (19–75)	63 (25–76)	.2	1.04 (1.004–1.081)	.03
Gender, female, No. (%)	15 (53.6)	22 (39.3)	.25		
AML diagnosis: relapsed/refractory, No. (%)	18 (64.3)	17 (30.4)	.005	4.55 (1.70–12.21)	.003
Chemotherapy, No. (%)					
3 + 7	6 (21.4)	20 (35.7)	.22		
FLAG	16 (57.1)	26 (46.4)	.49		
Clofarabine-based regimen	4 (14.3)	4 (7.1)	.43		
MEC	2 (7.1)	3 (5.4)	1.0		
Median baseline ANC on day 1 of chemotherapy	0.15	1.3	.14	0.96 (0.91–1.02)	.20
Baseline ANC <0.5, No. (%)	16 (57.1)	21 (36.2)	.10		
Diabetes, %	14.3	8.6	.47		
COPD, %	14.3	5.2	.43		
Current smoker, %	14.3	12.1	1.00		
Median duration of neutropenia before IMI, d	20	20	.43		
BMT before chemotherapy, %	10.7	8.9	1.00		
Risk factors in the previous 14 d					
Antifungal prophylaxis exposure, No. (%)					
Anti-*Aspergillus* azole	23 (82.1)	51 (87.9)	.52		
Voriconazole	14 (50)	27 (48.2)	1.00		
Posaconazole	10 (35.7)	25 (44.6)	.49		
Micafungin	9 (32.1)	18 (32.1)	1.00		
Antifungal prophylaxis exposure, relapsed/refractory AML patients, No. (%)					
Anti-*Aspergillus* azole	17 (94.4)	17 (100)	1.00		
Voriconazole	10 (55.6)	9 (52.3)	1.00		
Posaconazole	7 (38.9)	8 (47.1)	.74		
Micafungin	10 (55.6)	10 (58.8)	1.00		
ICU admission, %	21.4	10.7	.20	0.61 (0.15–2.46)	.48
Average ICU length of stay, d	6.5	7.9	.54		
Prolonged hyperglycemia, %	7.1	3.6	.56		

Abbreviations: 3 + 7, 3 days of daunorubicin plus 7 days of continuous infusion cytarabine; AML, acute myeloid leukemia; ANC, absolute neutrophil count; BMT, bone marrow transplant; CI, confidence interval; COPD, chronic obstructive pulmonary disease; FLAG, fludarabine, high-dose cytarabine, and granulocyte colony-stimulating factor; ICU, intensive care unit; IMI, invasive mold infection; LR, logistic regression; MEC, mitoxantrone, etoposide, and cytarabine; OR, odds ratio.

Antifungal prophylaxis exposure in the 14 days preceding IMI diagnosis (or matching date in the control group) did not differ between the IMI group and the non-IMI group (Table 2). When restricting the analysis to patients with relapsed/refractory AML, this distribution was also not different. Per EORTC/MSG criteria, of the 28 documented IFIs, 22 were classified as possible, 2 were probable, and 4 were proven. There were 2 cases of *Aspergillus versicolor*, 1 case of *Mucor* spp., and 1 case of *Fusarium* spp. The characteristics of patients with proven IMI are further displayed in Table 3.

**Table 3.** Characteristics of Proven Mold Infection Cases

Patient	Species	Source	Antifungal Exposure
1	*Aspergillus versicolor*	Bronchoalveolar lavage	Posaconazole
2	*Fusarium* species	Left thigh biopsy	Posaconazole and micafungin
3	*Mucor* species	Punch biopsy of skin	Voriconazole
4	*Aspergillus versicolor*	Sputum	Voriconazole

**Figure 2.**
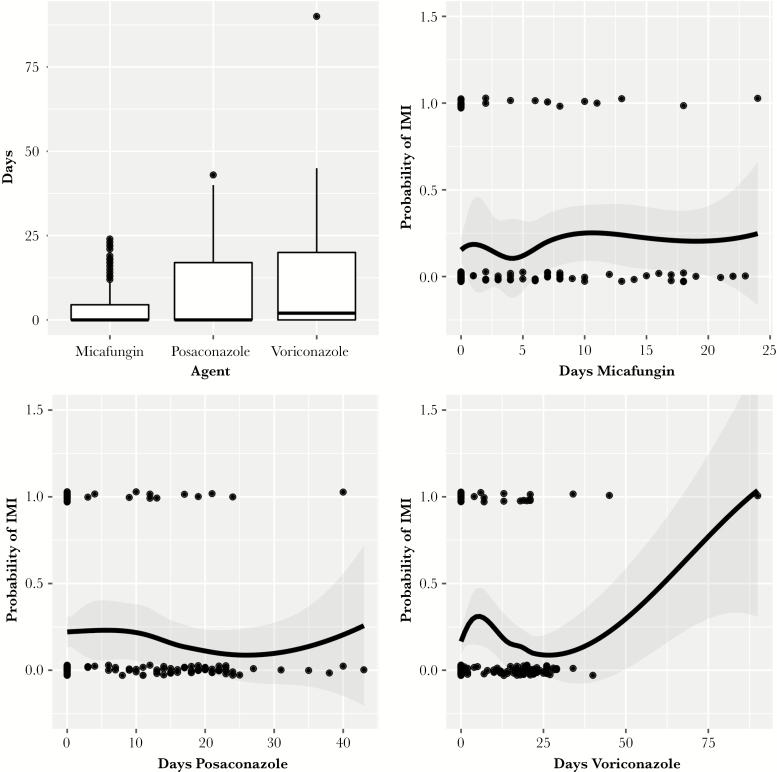
Impact of duration of prophylaxis on pppIMI incidence. The box-and-whisker plot shows the distribution of the duration of therapy for each agent. The LOESS plots are localized regressions that fit a smooth curve to the probability of having an IMI given the duration of therapy. The points mark whether a single observation had an IMI. Abbreviations: IMI, invasive mold infection; pppIMI, proven, probable, or possible invasive mold infection.

Variables with a *P* value ≤.2 that were included in the multivariable risk factor analysis were AML diagnosis (relapsed or refractory), age, baseline ANC before chemotherapy, and ICU admission. Of the variables evaluated, older age (*P* = .03) and relapsed/refractory AML (*P* = .003) were associated with an increased risk of IMI. The area under the curve (AUC) of the ROC curve of the model was 0.76 (95% confidence interval [CI], 0.619–0.857). Finally, both simple and multivariable logistic regressions did not show statistical significance in predicting IMI occurrence with the duration of therapy of the 3 antifungal prophylaxis agents (Figure 2; Supplementary Table 1).

## DISCUSSION

IMIs continue to remain a significant cause of morbidity and mortality in AML patients receiving RIC [1, 2, 12, 13]. Although anti-*Aspergillus* azole prophylaxis has made a significant impact in reducing the rate of IMIs in the AML population, the increasing use of oral targeted therapies in AML (eg, FLT3 inhibitors, IDH inhibitors, venetoclax), as well as therapies that predispose patients to veno-occlusive disease (eg, gemtuzumab ozogamicin), has forced many clinicians to increase utilization of echinocandins for prophylaxis. It is reassuring that our study demonstrated no significant increase in probable or proven IMI in patients receiving echinocandin prophylaxis. Given the rarity of ppIMI events, a potential limitation of our analysis was that it was underpowered to detect a true difference in ppIMI if one had actually existed between anti-*Aspergillus* azoles and micafungin; however, the fact that the detectable effect size calculated using sensitivity analyses was smaller than the difference between the groups in our data lends support to the claim that the nonsignificant *P* value is due to a true similarity between the groups rather than a lack of statistical power. It is also important to note that per-prophylaxis-day comparisons can be strongly influenced by duration of use and selection bias, which was the primary impetus for analyzing antifungal use in an adjusted model, which did not find the antifungal prophylaxis agent to be a significant risk factor for IMI in either univariable or multivariable analysis.

Although the rate of ppIMI was no different between anti-*Aspergillus* azoles and micafungin, the rate of possible IMI was higher in patients receiving micafungin prophylaxis. This may have been driven by a numerically higher proportion of relapsed/refractory patients receiving micafungin prophylaxis compared with anti-*Aspergillus* azoles (61% vs 41%, *P* = .065). Of note, relapsed/refractory AML was identified as an independent risk factor for IMI in our study. Additionally, the higher rate also may have been driven by a difference in practice management when faced with persistent fevers in the setting of nonspecific chest computed tomography (CT) findings in patients on echinocandin prophylaxis vs those on anti-*Aspergillus* azole therapy. Because of the historical concern for the possibility of breakthrough IMI in patients on echinocandin prophylaxis, most patients will be empirically switched to anti-*Aspergillus* azoles, without a well-established diagnosis, given the relative safety of switching to anti-*Aspergillus* azoles in this setting. However, in the exact same clinical scenario, patients on anti-*Aspergillus* azoles will often be maintained on therapy without declaring a nonspecific consolidation on CT of an IMI, as the alternatives either are more toxic (amphotericin B) or do not add significant additional *Aspergillus* coverage (switching anti-*Aspergillus* azoles). Thus, the retrospective nature of our study and potential over-reporting of possible IMIs in the echinocandin group may have contributed to the gap in the incidence of possible IMIs between the 2 groups.

To identify significant predictors of IMI in this at-risk population, a multivariable analysis was performed. The current multivariable model for predicting IMI has a high specificity and sensitivity, as indicated by the AUC of the ROC curve at 0.76. Because the incidence of ppIMI was low in our cohort, this risk factor analysis focused on assessing predictors for pppIMI. One advantage of our risk factor analysis was that we matched patients 2:1 based on the day in which IMI patients were diagnosed post-RIC, rather than examining risk factors that occur at any point during a patient’s remission induction course, which may occur after or well before presumed IMI diagnosis and could significantly confound such an analysis. To capture antifungal prophylaxis exposure that may have contributed to IMI development, rather than only examine the antifungal utilized on the specific date of IMI diagnosis, antifungals that were given for at least 96 hours in the 14 days preceding IMI diagnosis were counted as contributing to the development of the IMI. Importantly, on univariable analysis, there was no difference in micafungin use between the IMI and non-IMI groups (32.1% in both groups, *P* = 1.00).

In the multivariable model, patients with relapsed or refractory AML had a significantly higher risk of developing IMI. Other studies have also demonstrated a higher incidence of IMI in AML patients with relapsed or refractory disease [2, 14–17]. For instance, Lortholary et al. reported an almost 3-fold increase in the incidence of IA in patients with relapsed or refractory acute leukemia compared with patients receiving initial induction (67% vs 27%, *P* < .005) [16]. Patients with relapsed or refractory AML also tend to be neutropenic before treatment initiation; thus, the overall duration of profound neutropenia tends to be longer, significantly increasing the risk of development of IMI.

Although median baseline ANC was not significantly associated with IMI on multivariable analysis (OR, 0.96; 95% CI, 0.91–1.02; *P* = .19), this was numerically lower at baseline in patients who developed IMI (0.15 vs 1.3 × 10^9^ L^-1^), and the proportion of patients who were neutropenic at the start of chemotherapy was also numerically higher in the IMI group (57.1% vs 36.2%, *P* = .10). Duration of neutropenia has been identified in previous studies as a risk factor for IFI development [2, 4, 12, 18–22]. This factor was not significant in our analysis; however, we were limited in our ability to accurately capture the duration of neutropenia before the start of chemotherapy.

We also observed that increasing age was a significant predictor of the development of IMI, although the effect size was small (OR, 1.04; 95% CI, 1.004–1.081). Although the median age was numerically lower in the IMI group (58 vs 63 years, *P* = .2), there was a clear bimodal age distribution in the IMI group. Among the 6 patients with IMI in the younger “peak” of the bimodal distribution, all patients had relapsed or refractory disease; these outlier patients with this significant risk factor for IMI brought the median age in this group down. Advanced age has previously been reported as an independent risk factor for IFI in AML patients [14, 15, 23, 24]. Older patients often present with multiple comorbidities, worse performance status, and decreased bone marrow reserve, and are thus at higher risk for prolonged neutropenia and infectious complications from chemotherapy. Finally, older patients often present with high-risk AML features, including a higher proportion of unfavorable cytogenetics and secondary AML. This results in lower response rates, further increasing the incidence of relapsed/refractory disease and prolonged neutropenia in this population.

Interestingly, clofarabine, which has been previously identified as an independent risk factor, was not a significant risk factor for IMI in our study [7, 25]. Clofarabine is primarily used in older patients and in those with relapsed or refractory disease, and thus prolonged periods of neutropenia are likely the underlying risk factor for IFI. At our institution, use of clofarabine is relatively infrequent given the high toxicity rates of the regimen in less fit and older patients [26–28]. Accordingly, over the study period, use of clofarabine transitioned to being employed primarily in younger patients with very good performance status, which may help explain why clofarabine was not identified as an independent risk factor for IMI in our analysis.

These results are in contrast to the study by Gomes et al., which found an increased risk for fungal infections in patients on echinocandin prophylaxis. Major differences in study design may explain this discordance. Our study examined the IMI rate for each prophylaxis agent by only using days of therapy during the period of neutropenia or before discharge (median, 22 days), when the risk of IMI is greatest. The study by Gomes et al. examined the IFI incidence until day 120 after initiation of RIC. Because anti-*Aspergillus* azoles were continued beyond the period of neutropenia until up to day 120 and echinocandins—only available intravenously—were primarily utilized during the high-risk period of neutropenia, the denominator in the IFI per prophylaxis-day rate calculation was greatly changed, creating a bias against echinocandin therapy. Because of this, the lowest rate of IFI per prophylaxis-day was actually seen in patients receiving fluconazole prophylaxis, likely due to selection bias and use of this agent during lower-risk periods [29]. To further demonstrate the limitations of the IFI/IMI incidence calculation, the absolute numbers of ppIMIs and pppIMIs occurring on each agent in our study were similar (ppIMI: posaconazole 3, voriconazole 2, micafungin 3; pppIMI: posaconazole 12, voriconazole 15, micafungin 12); thus, the significant difference in the incidence rate of pppIMIs is driven largely by the overall lower use of micafungin at our institution (contributing to the lower denominator). To better assess the incidence density of IMI according to the prophylactic agent utilized, simple and multivariable logistic regressions examining the duration of prophylaxis on each antifungal agent as predictors of pppIMI were created. These models demonstrated no impact of duration of prophylaxis with each antifungal prophylaxis agent on pppIMI occurrence (Figure 2).

Many other differences exist between our study and that of Gomes et al. The breakthrough IFI rate in Gomes et al. was primarily driven by *Candida* spp., whereas our study focused exclusively on mold infections. The RIC regimens used at MD Anderson are more intensive (eg, CIA, FIA, FLAG-ida, and other investigational regimens) than the standard of care regimens most commonly used at our institution (primarily 7 + 3 or FLAG), which could have increased the IFI rate in Gomes et al. [25, 30–32]. Finally, our prophylaxis strategy with echinocandins is different as we utilize higher doses of micafungin (100 mg vs only 50 mg). Recently, a randomized controlled trial in acute leukemia and myelodysplastic syndrome patients compared micafungin 100 mg with posaconazole suspension as antifungal prophylaxis during the period of neutropenia [8]. Similar to our study, the rate of proven or probable IFI during the prophylaxis period was low in this analysis, and there was no significant difference between the 2 groups (3.4% micafungin vs 1.7% posaconazole) [8]. There are several limitations to our study that should be addressed. The choice of antifungal agent was at the discretion of the primary team. Thus, there is a possibility for selection bias. Similar to the study by Gomes et al., use of micafungin was limited and primarily restricted to the high-risk period when patients had contraindications or intolerance to anti-*Aspergillus* azoles. For this reason, the total days of micafungin prophylaxis was 554 days compared with 1702 days and 1374 days for voriconazole and posaconazole, respectively. The significantly lower denominator in the micafungin group may have impacted the per-prophylaxis-day comparison and inflated the IMI PPD rate in the micafungin arm. Because this was a single-center study, risk factors identified at Michigan Medicine may differ from those at other institutions, including those that use different induction and re-induction chemotherapy strategies that may be more toxic, and may not be generalizable. Finally, although assessing antifungal prophylaxis and other risk factor exposures in the 14-day period before IMI development (or corresponding matching day in the control patients) was used to more accurately capture risk factors contributing to IMI development, the time points chosen (minimum 96 hours of antifungal use during the preceding 14 days) were based on a consensus from our study group but have not been evaluated previously, which represents a possible limitation of such an analysis. Additionally, because the number of pppIMI events was low at 28 patients, there is the possibility for introduction of bias into the multivariable model, which included 4 covariates, although it is reassuring that the risk factors identified in our model were consistent with prior studies. As previously mentioned, unlike other studies, we focused only on IMI, given the significant morbidity and mortality associated with mold infections and based on recent literature that has demonstrated a shift in IFIs in AML patients from yeast to mold infections with the advent of azole prophylaxis [2, 4, 13, 33–35]. The rate of breakthrough invasive candidiasis/candidemia is very low in AML patients post-RIC at our institution; thus we felt this may confound our results.

In conclusion, our study demonstrated no difference in the rate of ppIMIs between patients receiving anti-*Aspergillus* azoles and those receiving micafungin 100 mg once-daily prophylaxis. With the approval of several new targeted therapies that pose a significant risk for drug interactions or concomitant hepatotoxicity during RIC for AML, clinicians can feel more assured with the use of echinocandins for antifungal prophylaxis in this setting. Older patients and patients with relapsed/refractory AML were associated with the development of IMI on multivariable analysis despite antimold prophylaxis; thus, caution and close monitoring are warranted in this population based on this association.

## Supplementary Data

Supplementary materials are available at *Open Forum Infectious Diseases* online. Consisting of data provided by the authors to benefit the reader, the posted materials are not copyedited and are the sole responsibility of the authors, so questions or comments should be addressed to the corresponding author.

ofz176_suppl_supplementary_figure_1

ofz176_suppl_supplementary_table_1
