# Supplementary material for: Incidence and Risk Factors for Breakthrough Invasive Mold Infections in Acute Myeloid Leukemia Patients Receiving Remission Induction Chemotherapy
Source: Open Forum Infect Dis. 2019 Apr 12;6(5):ofz176. doi: 10.1093/ofid/ofz176 (PMC6524834; doi:10.1093/ofid/ofz176)

Supplemental Figure 1: Risk factor analysis method. Patients with IMI were matched 2:1 to patients without IMI. Risk factor analysis is based on the 14 days preceding IMI diagnosis. RIC, remission induction chemotherapy, IMI, invasive mold infection, D_0_, date of IMI diagnosis, D_-14_, start of risk factor analysis period.


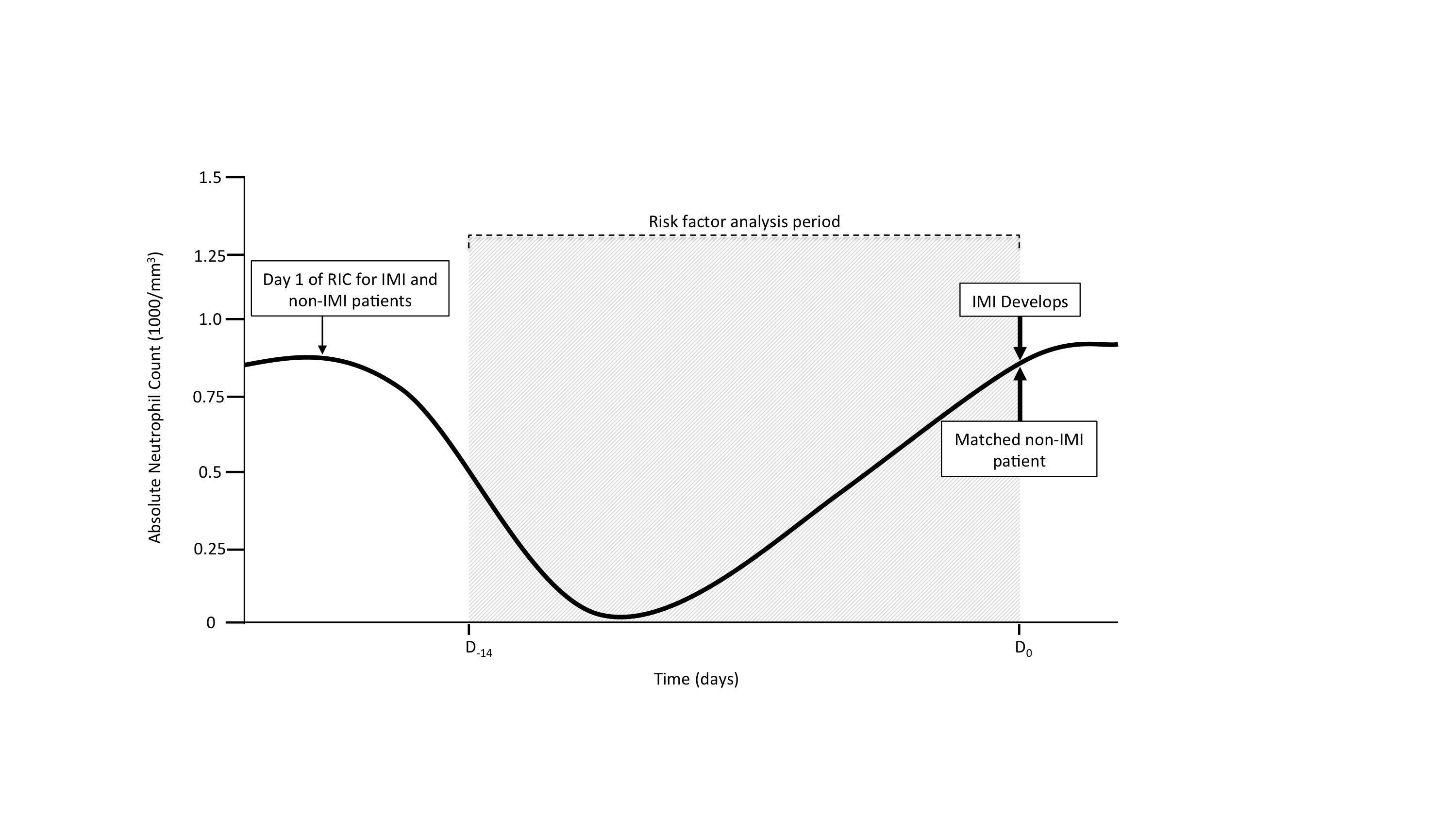

Supplement: ofz176_suppl_supplementary_figure_1 [file ofz176_suppl_supplementary_figure_1.docx]
