# Supplementary material for: Incidence and Risk Factors for Breakthrough Invasive Mold Infections in Acute Myeloid Leukemia Patients Receiving Remission Induction Chemotherapy
Source: Open Forum Infect Dis. 2019 Apr 12;6(5):ofz176. doi: 10.1093/ofid/ofz176 (PMC6524834; doi:10.1093/ofid/ofz176)
Supplement: ofz176_suppl_supplementary_table_1 [file ofz176_suppl_supplementary_table_1.docx]

**Supplemental Table 1: Logistic regression of duration of prophylaxis on pppIMI occurrence**

| Variable | OR | 95% CI | p-value |
| --- | --- | --- | --- |
| Intercept | 0.18 | (0.10, 0.30) | <0.001 |
| Voriconazole | 1.01 | (0.98, 1.04) | 0.432 |
|  |  |  |  |
| Intercept | 0.28 | (0.17, 0.46) | <0.001 |
| Posaconazole | 0.97 | (0.93, 1.01) | 0.24 |
|  |  |  |  |
| Intercept | 0.19 | (0.12, 0.30) | <0.001 |
| Micafungin | 1.02 | (0.95, 1.09) | 0.526 |
|  |  |  |  |
| Intercept | 0.24 | (0.10, 0.61) | 0.002 |
| Voriconazole | 1.01 | (0.97, 1.04) | 0.696 |
| Posaconazole | 0.98 | (0.93, 1.03) | 0.496 |
| Micafungin | 1.01 | (0.94, 1.08) | 0.796 |
